# Elemental analysis and micromorphological patterns of tooth/restoration interface of three ion-releasing class V restorations

**DOI:** 10.1186/s12903-024-04944-w

**Published:** 2024-10-15

**Authors:** Hebatallah Sarhan, Rabab Mehesen, Hamdi Hamama, Salah Hasab Mahmoud

**Affiliations:** 1 Conservative Dentistry Department, Faculty of Dentistry, Mansoura University, Mansoura, Egypt; 2 Restorative Dentistry Department, Faculty of Oral and Dental Medicine, Delta University for Science and Technology, Gamasa, Egypt

**Keywords:** EDX, SEM, Bioactive materials, Ion-release, Giomer, RMGI

## Abstract

**Objectives:**

To evaluate and compare the ion-releasing capability of three different restorative systems at the restoration/tooth interface elemental analysis using energy-dispersive X-ray technique. Additionally, micromorphological patterns of the restoration/tooth interfaces was investigated.

**Materials and methods:**

Eighteen freshly extracted sound human premolars were collected for the study. The premolars were randomly assigned into 3 groups (*n* = 6) based on the type of restorative materials used: Giomer (Beautifill II), ion-releasing composite (Activa Presto), and RMGI (Riva Light Cure). Half of the specimens in each group were tested after 24 h (the “immediate group”), while the remaining half were tested after 6 months of storage in deionized water (the “delayed group”). Standardized box-shaped cavities along the cervical area of teeth crowns and restored them with the assigned restorative material following manufacturers’ instructions. The specimens were sectioned buccolingually into 2 halves. One half of each specimen was subjected to elemental analysis using energy-dispersive X-ray technique (EDX), while the remaining half was sputter coated and underwent micromorphological analysis of the restoration/tooth interface using a scanning electron microscope (SEM). The collected data from elemental analysis test were tabulated and subjected to statistical analysis.

**Results:**

The two-way ANOVA test showed significant differences in both phosphorus and calcium levels among the tested restorative systems (*p* < 0.05). In the immediate subgroup, RMGI recorded the highest phosphorus level (0.1527), followed by the ion-releasing composite (0.1172), while Giomer exhibited the least levels (0.0326) (*p* < 0.05). The ion-releasing composite group had the highest calcium level (0.2797), followed by RMGI (0.248), and Giomer (0.2385) respectively (*p* < 0.05). In the delayed subgroups, Giomer recorded the highest phosphorus level (0.1526), followed by the ion-releasing composite (0.1058), and RMGI group (0.0466) respectively (*p* < 0.05). RMGI had the highest calcium level (0.2801), followed by the ion-releasing composite (0.2659), and Giomer had the lowest level (0.1792) (*p* < 0.05). The micromorphological analysis of the restoration/tooth interfaces showed good adaptation between the composite and tooth substrate in different restorative groups.

**Conclusions:**

The ion-releasing capability of the three restorative systems appears to be comparable. The rate of mineral release and diffusion is affected by time and composition.

**Supplementary Information:**

The online version contains supplementary material available at 10.1186/s12903-024-04944-w.

## Introduction

Bioactive materials are constantly being introduced into the dental market to improve the properties of currently available restorative materials. Bioactive materials can be defined as materials that have the ability to elicit a response from living tissue, such as inducing hydroxyapatite formation [1]. The development of therapeutic bioactive materials with favorable ion release may reduce the caries rate at restoration margins and provide protection for these areas that are prone to secondary caries. This can be achieved by facilitating remineralization and restoring tooth mechanical properties [2].

The release of fluoride, calcium, and phosphate ions is essential characteristic for future restorative materials [3]. These ions can affect microbial metabolism and enhance the deposition of apatite-like compounds at tooth/restoration interface. Consequently, this may lead to a significant increase in tooth resistance to acid attacks and improving longevity of restorations [4]. Additionally, bioactive materials are characterized by their ability to respond to environmental changes. In the circumstance of intra-oral pH drop, bioactive materials release hydroxyl ions to neutralize the acid created by the biofilm and alkalize the environment. This might help in preventing tooth demineralization and enhance tooth remineralization [5].

Giomer, ion-releasing composite, and RMGI are examples of currently available bioactive restorative materials. RMGI is categorized as biocompatible restorative material and its bioactivity is attributed to their ability to crystallization with tooth substrate in the presence of water. This generates fluoro-apatite crystals that are more acid resistant than hydroxyl-apatite crystals. Giomer is a combination of glass ionomer and composite, sometimes it is referred to as a ‘hybrid composite’ [6]. The manufacturing technology of this material depends on incorporation of pre-reacted glass (PRG) filler particles into a resin matrix [7].Therefore, Giomers exhibit properties of both glass ionomers as well as resin composites [8]. They are similar to RMGI and resin composites in that they are light-activated and require the use of a bonding agent to adhere to tooth structure [9]. Hence, this increases the demand for development of alternative smart restorative materials, such as ion-releasing composite. These materials are claimed to stimulate hydroxyapatite formation and induce remineralization at the tooth/restoration interface by releasing significant amounts of calcium, phosphate, and fluoride ions over time [10]. These materials are climed to be moisture-friendly and releases fluoride ions similar to RMGI and Giomer [11]. It is hypothesized that bioactivity of these restorative materials helps in hermetically sealing restoration margins and might improve the durability of te restorations [12].

The remineralization potentials can be assessed using energy dispersive x-rays (EDX) method [2]. This non-invasive method has the ability to elementely analyze the composition of the tested material specimens [13]. In this study EDX was used to assess the ion-releasing capability of the tested materials [14]. Furthermore, it is beneficial to evaluate the durability and stability of the interface between the tooth structure and the restoration by using micromorphological characterization of the restoration/tooth interface with SEM [15]. Failures at the tooth/restoration interface might lead to creation of marginal gaps allowing microbes, toxins, and food debris penetration at interfacial micro-gaps [16].

There are few studies providing consistent guidance on the protentional remineralizing capabilities of Giomers and ion-releasing composites. Therefore, the objective of the current study was to evaluate and compare the release of phosphorus and calcium ions at the tooth/restoration interface of Giomer, ion-releasing composite, and RMGI. The effect of storage on the rate of ion release was also assessed using EDX and SEM. Hence, the null hypothesis of the study was that there would be no difference in ion release and marginal adaptation among the three restorative systems.

## Materials and methods

Materials tested in the current study were Giomer (Beautfill II Shofu Dental, GmbH, Japan), ion-releasing composite (Activa Presto Pulpdent, USA) and RMGI (Riva Light Cure SDI, Australia). The materials were used according to the manufacturer’s instructions and the full description of the materials is illustrated in Table 1.

**Table 1:** Material used in the study

Material	Type	Composition	Manufacture
Scotchbond Etchant	Etching Gel	phosphoric acid 37%	3 M ESPE, St Paul, MN, USA
Single Bond Universal	Universal Adhesive	MDP phosphate monomer, dimethacrylateresin, HEMA, filler, ethanol, water, initiator, silane, vitrebond copolymer.	3 M ESPE, St Paul, MN, USA
Beautifil II	Giomer	fluoride-releasing, nanohybrid direct aesthetic restorative material that contains the modified S-PRG fillers	Shofu Dental, GmbH, Japan
Activa Presto	ion – releasing composite	Mineral enriched composite that releases and recharges calcium, phosphate, and fluoride.	Pulpdent, USA
Riva Conditioner	Polyacrylic Acid Conditioner	Polyacryl acid 25–30% by wt Balance ingredient (non-hazardous) 70–75% wt	SDI, Australia
Riva Light Cure	Resin Modified Glass Ionomer	2-hydroxyethyl methacrylate, acrylic acid homopolymer, dimethacrylate crosslinker, acidic monomer, tartaric acid, glass powder.	SDI, Australia

### Tooth selection

A total number of eighteen extracted human permanent premolars were collected from Oral Surgery Department Clinic, Faculty of Dentistry, Mansoura University. These premolars had been extracted for orthodontic reasons. The Ethical Committee of the Faculty of Dentistry, Mansoura University approved the study protocol and procedures under approval #A04010720.

A hand scaler (Nordent, Ivory #2–3, USA) was used to remove any hard deposits or remnants of soft tissue from the specimens. Then, they were rinsed with running water and disinfected by of storage in a 0.5% chloramine-T solution for 48 h [17]. A stereomicroscope (Olympus model SZ-PT, Tokyo, Japan) was used to examine cracks or fractures and any cracked premolars were discarded. The specimens were then polished by using slurry of pumice (PSP, Dylan Rd, Belvedere, England) and rubber cup (prophy rubber polishing cup, China). They were then kept in deionized water, which was changed every five days, at 4 °C ± 1 °C until they were needed.

### Study design and specimen preparation

A standardized box-shaped cavity (3 mm occlusogingival × 4 mm mesiodistal × 2 mm deep) was prepared along the cervical area of the buccal surface of all teeth using straight abrasive tip (6836 kR.314.014; Komet, Brasseler GmbH Co. KG, Lemgo, Germany) installed to high speed hand-piece (Sirona T3, Bensheim, Germany) under copious air-water cooling. A digital caliper was used to measure the length and width, while an inerasable pen was used to sketch the dimensions and create cavities [17]. Furthermore, the cavity’s depth was checked using the bur’s stopper and evaluated on a regular basis using a periodontal probe [18].

Using random assignment, specimens were divided into three groups based on type of restorative materials used; Giomer (Beautifill II), ion releasing composite (Activa Presto), or RMGI (Riva Light cure) (*n* = 6). Each group was further sub divided into 2 subgroups based on its time of storage; Subgroup I (*n* = 3) tested immediately while subgroup II (*n* = 3) tested after 6 months of storage in deionized water.

Prior to restoring the Giomer group and ion-releasing composite group, the enamel was selectively etched for 30 s with 37% phosphoric acid gel (Scotchbond Etchant, 3 M ESPE, St Paul, MN, USA). The preparation was then completely washed with water for 10 s, gently air dried, and a universal bonding agent (Single Bond Universal, 3 M ESPE, St. Paul, MN, USA) was applied to the etched enamel and dentin surface using micro brush for 20 s. To evaporate the solvent, the bonding agent was gently air dried for 5 s before being light cured for 10 s with a regulated light emitting diode (LED) curing device (Elipar S10, 3 M ESPE, USA). This light curing unit has a wave length of 430–480 nm and a light intensity of 1200 mW/cm2, which was monitored regularly during the restorative procedures with a radiometer (Bluephase Meter, Ivoclar Vivadent).

Giomer and ion-releasing composite were applied incrementally (2 mm thickness) to the prepared cavities with a gold plated instrument and light cured for 20 s with (LED) curing device (Elipar S10, 3 M ESPE, USA). The restorations were then shaped and finished using a high-speed diamond finishing instruments (4092.314, Komet) under copious air-water cooling. In order to achieve a smooth surface, a composite polishing kit (Shofu Inc, Kyoto, Japan) and Sof-lex discs (3 M ESPE, ST. Paul, MN, USA) were used in sequence; Course, medium, fine, and superfine.

Regarding RMGI group, dentin conditioning was initially applied using 25–30% polyacrylic acid (Riva Conditioner, SDI, Australia) for 10 s. The conditioner was then completely washed with water, and excess water was air dried to keep the dentin wet. RMGI was then added to the cavity in increments of no more than 2 mm into the cavity, shaped and light cured for 20 s with (LED) curing device (Elipar S10, 3 M ESPE, USA). The RMGIC specimens were coated with surface coat (Riva Coat, SDI, Australia) following the manufacturer’s instructions.

**Fig. 1:**
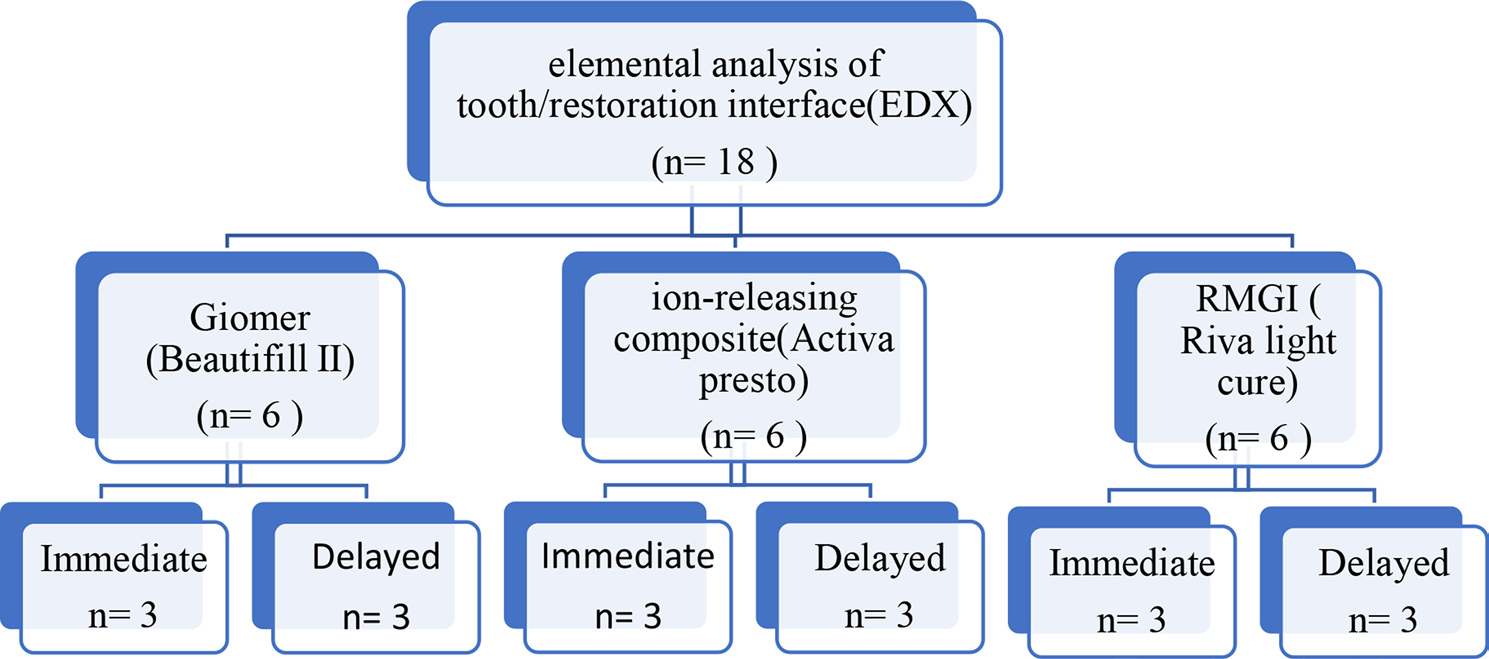
Schematic diagram showing specimens grouping in elemental analysis of tooth / restoration interface using EDX

After restoration, each group further subdivided into two subgroups: subgroup I (*n* = 3) tested immediately after 24 h of storage. While, subgroup II (*n* = 3) tested after 6 months of storage. Specimens stored in deionized water in a tight-seal plastic jar labeled according to restoration type as shown in Fig. 1.

Every specimen was standardized by mounting it vertically in polyvinyl chloride rings (PVC, 1.4 × 2.5 cm). The rings were then filled with auto-polymerizing acrylic resin (Acrostone, Acrostone Dental Factory, Egypt), after the acrylic resin had set; the specimens were removed from the PVC molds, producing a block. As a result, the specimen can be sectioned standardized with a slow speed diamond saw (ISOMET 4000; Buehler, Lake Bluff, IL, USA) under water cooling system. Each specimen sectioned longitudinally through the restoration’s center into two halves in buccolingual direction. One half used for EDX elemental analysis and the other half used for micromorphological analysis. The cut surfaces were polished with 400, 600,1200, 2000, and 4000 grit silicon carbide papers (MicrocutTM, Buehler, Lake Bluff, IL, USA) followed by 1 μm diamond pastes (Diamat, Pace Technologies, Tuscon, AZ, USA) and the surfaces were then ultrasonically cleaned (CD-4820 ultrasonic cleaner, Codyson, Shenzhen, China) for 1 min in deionized water [19].

### Elemental analysis with energy-dispersive X-ray (EDX)

One half of each specimen was analyzed using EDX. The selected halves were analyzed using high vacuum field emission microscopy. Images were captured using a secondary electron detector (OXFORD X MAX 20, USA) at a distance of 100 μm, with a high voltage of 20 kV and magnification of 1000×. The EDS spectrometer was connected to a scanning electron microscope (JSM-6510LV SEM, JEOL Ltd, Tokyo, Japan) to quantitatively assess restoration/tooth interface of each specimen. A histogram plot was generated by the EDX detector to illustrate the calcium and phosphate weight percentages.

### Micromorphological analysis of restoration/tooth interface under SEM

The second half of each specimen was prepared for the acid-base challenge by first etching them with a 10% orthophosphoric acid solution for 10 s, rinsing them with water for 10 s, and then applying 5% sodium hypochlorite (NaOCl) for 5 min [20]. The samples were dried and mounted on aluminum stubs and then sputter coated with gold alloy by using a gold sputter coating apparatus (SPI Module - Sputter Carbon / Gold Coater, EDEN instruments, Japan). The specimens were then inspected at tooth/restoration interface using scanning electron microscope (JEOL JSM 6510 lv, Japan) at 20 kv and working distance of 10 μm. Micrographs at magnifications of 2000 × were taken.

### Statistical analysis

All the collected data was tabulated using Microsoft excel then subjected to statistical analysis using SPSS software, version 25 (SPSS Inc., PASW statistics for windows version 25. Chicago: SPSS Inc.). Normality tests were conducted to select the suitable statistical tests.

Quantitative data were described using mean ± Standard deviation for normally distributed data after testing normality using Shapiro Wilk test. Significance of the obtained results was judged at the (≤ 0.05) level. Two Way ANOVA test was used to study the combined effect of 2 independent factors on dependent continuous outcome.

## Results

### Elemental analysis of restoration/tooth interface using energy dispersive X-ray (EDX)

**Fig. 2:**
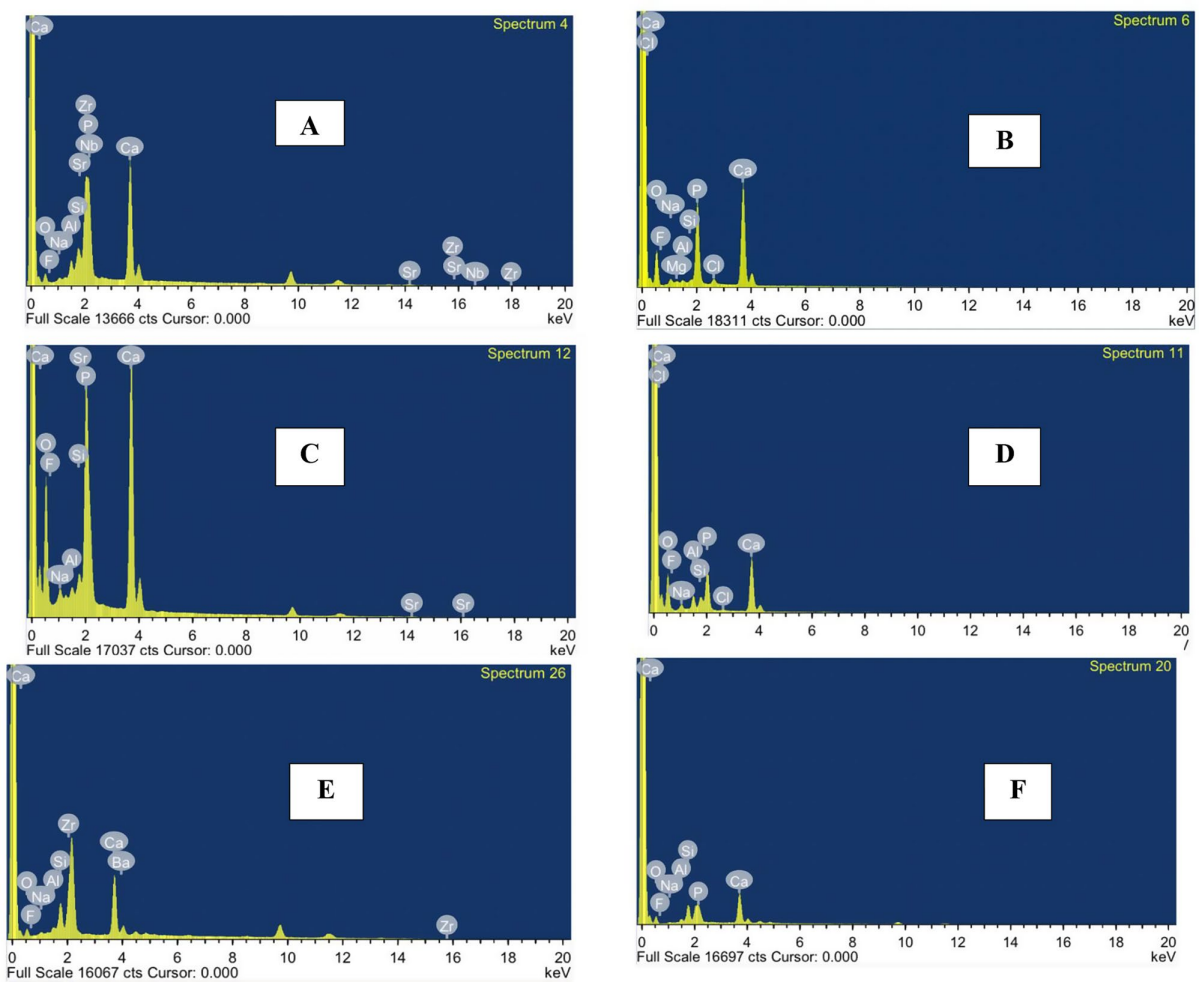
EDX elemental analysis at tooth/restoration interface (**A**) Giomer immediate analysis, (**B**) Giomer delayed analysis, (**C**) ion-releasing composite immediate analysis, (**D**) ion-releasing composite delayed analysis, (**E**) RMGI immediate analysis, (**F**) RMGI delayed analysis

The mean squares of the specimens in the three different groups at different times (immediate and delayed) are shown in Table 2. The ion exchange at restoration tooth /interface was shown on EDX mapping (Fig. 2).

For immediate measurements; (0.1527) the highest phosphorus level was detected in RMGI followed by (0.1172) ion-releasing composite while the least (0.0326) was detected in Giomer (*p* < 0.05). The highest calcium level (0.2797) was recorded in ion-releasing composite group followed by (0.248) RMGI while the least (0.2385) was recorded at Giomer (*p* < 0.05).

For delayed measurements; (0.1526) the highest phosphorus level was detected in Giomer followed by (0.1058) ion-releasing composite and the least (0.0466) was recorded at RMGI group (*p* < 0.05). The highest calcium level (0.2801) was detected among RMGI followed by (0.2659) ion-releasing composite and (0.1792) Giomer (*p* < 0.05).

Two Way ANOVA test revealed the effect of changing in study group and time of assessment and the combination between 2 factors on phosphorus level and on calcium level (*p* < 0.05). A statistically significant effect is detected for change in time and group and the combined effect of both factors (*p* < 0.05). Tukey Post Hoc multiple comparison test was used to assess pairwise for phosphorus level and for calcium level comparison between subgroups and demonstrates that between ion-releasing composite and Giomer (*p* < 0.05), between ion-releasing composite and RMGI (*p* < 0.05), and between RMGI and Giomer (*p* < 0.05).

**Table 2:** Comparison of calcium and phosphorus levels between Giomer, ion-releasing composite and RMGI at tooth / restoration interface, p1: difference by ion-releasing composite and RMGI groups, p2: difference between ion-releasing composite and Giomer groups, p3: difference between RMGI and Giomer groups by post Hoc Tukey test

		ion-releasing composite	RMGI	Giomer	test of significance #	Within group significance
Phosphorus	Immediate	0.1172 ± 0.0002	0.1527 ± 0.0001	0.0326 ± 0.00017	*p* < 0.001*	p1 < 0.001* p2 < 0.001* p3 < 0.001*
	Delayed	0.1058 ± 0.0002	0.0466 ± 0.00015	0.1526 ± 0.0001	*p* < 0.001*	p1 < 0.001* p2 < 0.001* p3 < 0.001*
Calcium	Immediate	0.2797 ± 0.0002	0.248 ± 0.0001	0.2385 ± 0.0001	*p* < 0.001*	p1 < 0.001* p2 < 0.001* p3 < 0.001*
	Delayed	0.2659 ± 0.025	0.2801 ± 0.0001	0.1792 ± 0.0002	*p* < 0.001*	p1 < 0.001* p2 < 0.001* p3 < 0.001*

### B. Micromorphological analysis using scanning electron microscope (SEM)

**Fig. 3:**
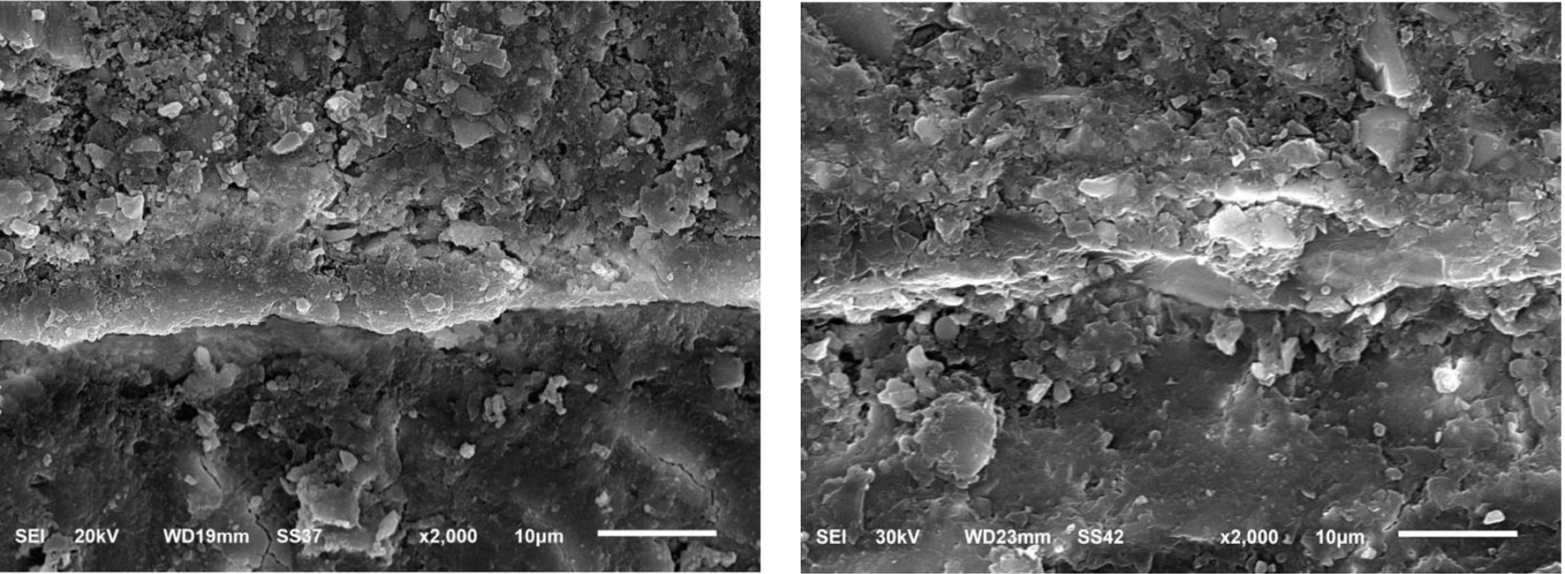
SEM micrography showing micromorphology of tooth/ giomer interface: (**A**) immediate scanning, (**B**) delayed scanning

**Fig. 4:**
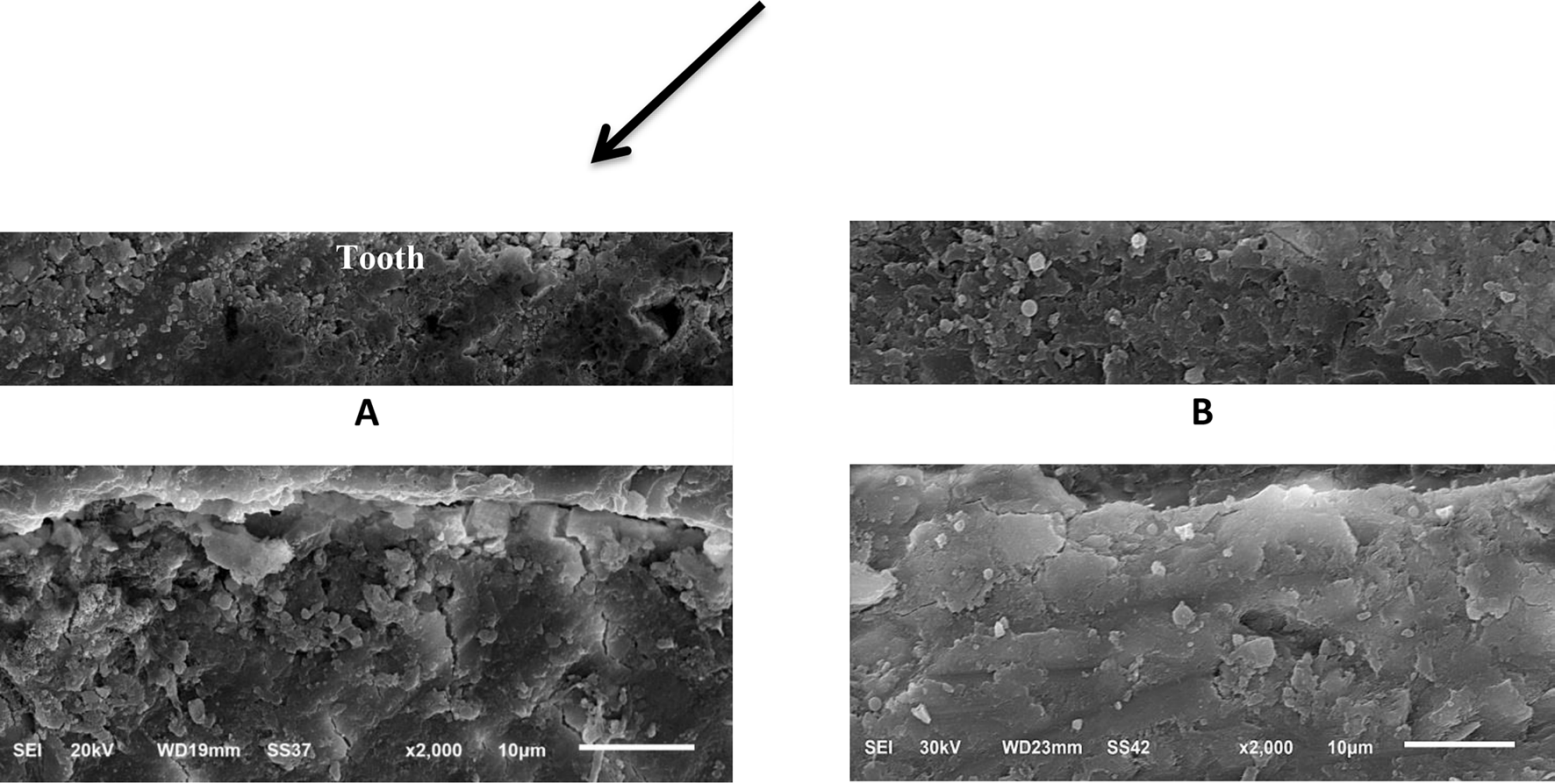
SEM micrography showing micromorphology of tooth/ ion-releasing composite: (**A**) immediate scanning, (**B**) delayed scanning (the arrows pointing to crystal-like structure)

**Fig. 5:**
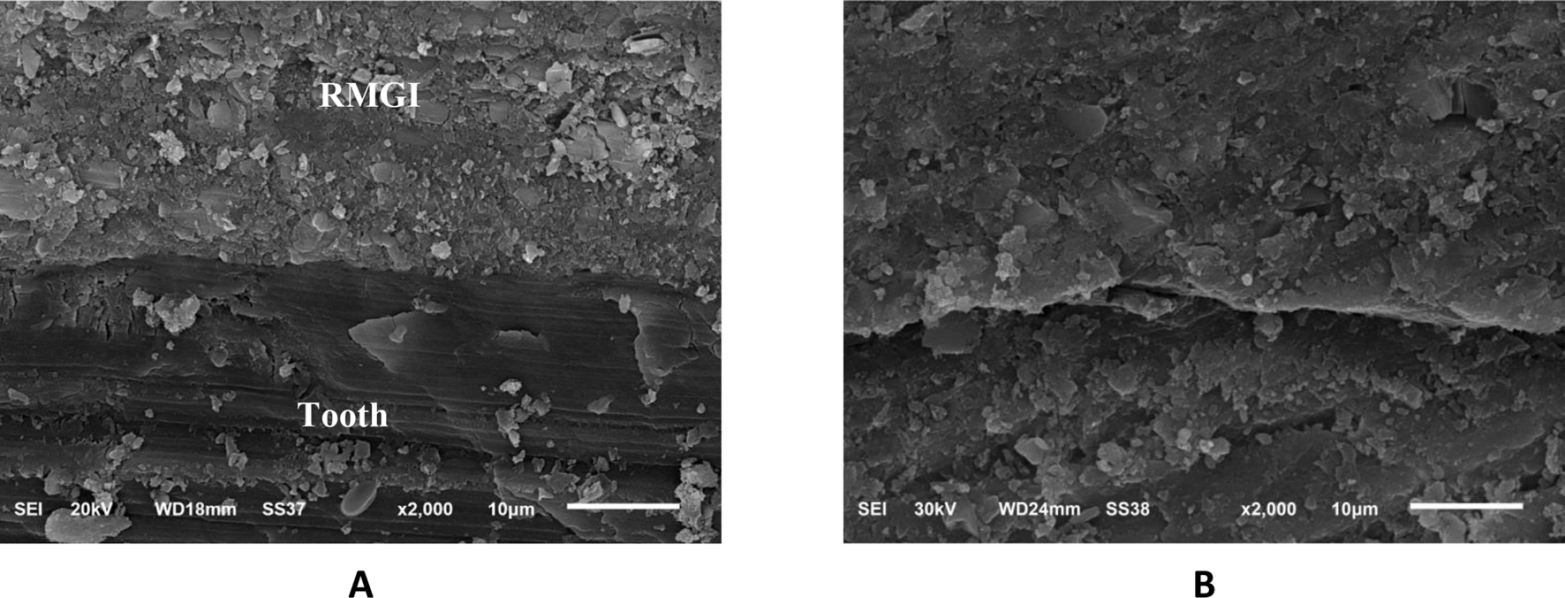
SEM micrography showing micromorphology of tooth/ RMGI interface: (**A**) immediate scanning, (**B**) delayed scanning

A descriptive micromorphological analysis of the interfaces between different restorations and the tooth structure was carried out for each group immediately and after 6 month storage in deionized water (Figs. 3 and 4, and 5). The immediate SEM images in Fig. 3-A at magnification (× 2000) revealed that Giomer / tooth interface showed good adaptation, continuous interfaces between the restoration and tooth substrates. It showed large filler particles with varying sizes, show denser particle and little voids than RMGI and ion-releasing composite and a relative degree of polymer matrix swelling can be seen in delayed Giomer micrographs Fig. 3-B.

The observation of delayed ion-releasing composite groups revealed tightly sealed interface associated with crystal-like depositions in delayed groups (Fig. 4-B). Moreover, the micromorphological patterns RMGI/dentin interface showed intimate adaptation to the underlying tooth structure indicating the formation of hybrid-like layers (Fig. 5).

All tested materials/tooth interfaces revealed the development of a hybrid-like layer and demonstrated interdiffusion zone or ion exchange layers, which were more apparent after time of storage. Also, the interfaces showed unnoticeable changes after aging in delayed SEM micrographs (Figs. 3-B and 4-B, and 5-B).

## Discussion

It is important to select a bioactive restorative material to restore class V lesions that cause a local increase in calcium and phosphate ions and have the potential to alkalize the oral environment which is a promising method to enhance remineralization and prevent secondary caries [3, 16]. The current study was conducted to evaluate ion-releasing capability at restoration/tooth interface of three different restorative systems. Smart restorative material described by the manufacturer as an ion-releasing composite [10], was selected to evaluate its calcium and phosphorous ions releasing property and its claimed bioactivity. Hence, RMGI was selected to experimental comparison to ion-releasing composite. Also, it is compared with Giomer which is a combination of RMGI and composite to give properties of both glass ionomers and resin composites. As well as Giomer can stimulate remineralization and inhibit caries formations [21]. This provides a fair comparison to this bioactive restorative material as these materials have similar clinical indications and related compositions, which makes them comparable, enabling practitioners to assess prognosis of the restorative material [2].

Elemental analysis is a technique used to detect the production of apatite containing calcium and phosphorus on the surface of bioactive materials [22]. Ions release determination was typically used to evaluate the remineralization process [23]. Moreover, EDX software offers a “zone selection” option; it is a reliable tool for identifying minute variations in mineral composition between confluent areas [20]. Furthermore, SEM was used for examining the restoration/tooth contact is a typical technique for characterization [16, 24]. Despite the fact that the results of this classic observation approach provide significant information about the tooth/ restoration interface, a number of difficulties could compromise its accuracy, when the sample’s water content quickly evaporates inside SEM vacuum chambers, it causes microcracks and deponding of the restoration/tooth interface. Additionally, the ion-exchange between the restorative material and the tooth structure cannot be accurately determined using SEM [16]. That`s why, in the current study each tooth of each group sectioned longitudinally through the restoration’s center into two halves in buccolingual direction [17, 22], one half used for micromorphological analysis using SEM and the other half used for EDX elemental analysis, reducing the occurrence of cracks in the restorative material inside the SEM chamber. The use of deionized water in this study for samples storage was chosen to ensure that there was no interaction between storage medium and specimens, due to the absence of inorganic “minerals” like calcium, phosphorus, and magnesium in distilled water [25].

The amount of phosphorus levels was significantly greater for immediate measurements at tooth/restoration interface than delayed measurements and this may be due to the burst release of ions in case of RMGI and ion-releasing composite [26, 27]. Burst release of ions in the RMGI is due to the superficial dissolution of this material. However, this dissolution decelerates by time. So, ion release from the mass material into the environment decreases in delayed measurements [26]. As well as, the high-water sorption of RMGI at the onset of acid-base reactions and high speed of reactions, causing the release of higher amounts of phosphate ions compared with other materials [3, 27]. In addition, the high porosity of RMGI materials may allow greater ion release [3].

Ion-releasing composite diffused small amounts of phosphate ions than RMGI for immediate measurements, which might be attributed to the formation of a superficial layer of calcium fluoride and calcium phosphate, which characterized with slow dissolution [3]. Also, this observation was explained by Garoushi et al. [28]who reported that the release of ions from RMGI was greater than that of ion-releasing composite in the first 24 h, and this consistent with our findings. On the other hand, delayed measurements of ion-releasing composite were higher for phosphorus ion which was consistent with the study conducted by Kasraei et al. [3] and Sajini et al. [2] Who revealed that ion-releasing composite release phosphate ions more than glass ionomer over time.

On the contrary, Giomer based restorative system did not provide the same burst release and it showed the least phosphorus level for immediate measurement and the highest for delayed measurements. The difference of ion release tendency could be due to Giomer showed the lowest water sorption probably due to the high content of filler particles 83.3 wt%, the presence of surface pre-reacted glass-ionomer particles in the Giomer [29, 30]. Therefore, water sorption does not contribute significantly to phosphorous ion release, and according to Study conducted by Gonulol et al. [31], stipulated that ion release from a restorative material is known to be mediated by its water diffusion ability. Additionally, aging is thought to have changed the material surface and opened porosities for ions to accumulate and then release. These factors could explain why phosphorus delayed measurements for Giomer significantly increased as our specimens were already aged for 6 months. The current study results were consistent with the studies of a Kelić et al. [32] and Francois et al. [6] who reported that Giomer have been demonstrated to have a very low ion release that is significantly lower than RMGI and this may be due to a greater porosity of glass ionomer, Giomer hydrophobic resinous matrix and it have low amount of pre-reacted glass ionomer fillers. There is a shortage in published studies related to studying the release of calcium and phosphate ions from Giomer restoration [2], except study conducted by Alinda et al. [33] who compared EDX analysis of GIC and Giomer; as both have the same chemical elements and release calcium and phosphorus. Also, the study conducted by Sobh et al. [34] revealed that Giomer releases calcium and phosphorous ions. Thus, the release of calcium and phosphate ions from Giomer restoration may be attributed to its composition as contains pre-reacted glass-ionomer (PRG) particles [35] .

The increase in calcium ions was significant in contrast to the increase in phosphorus ions for the three different materials; this finding could be a result of the high filler content of these materials [19]. The calcium ion release was found to be significantly high for ion-releasing composite compared to RMGI and Giomer. The observed variations in calcium ion release can be linked to the variations in their compositions.

The calcium ion level of ion-release composite can be attributed to its bioactive fillers and unique bioactive ionic resin, which is responsible for significant calcium release and recharge [22]. Furthermore, the absence of Bis-GMA in the composition facilitated improved ion release [22]. Puspitasari et al. [36] reported that bioactive glass; which is made of silica (SiO2), phosphorus pentoxide (P2O5), and boron trioxide (B2O3); is included in the bioactive resin. Water molecules can more easily infiltrate the resin because the silica network in bioactive glass is more open to create silanol or Si(OH)4 due to hydrolyzed silica, water molecules that penetrate the silica glass may come into touch with hydronium ions (H+), modifier ions Na+, and Ca2+ [36]. This ion exchange process then takes place. In order to create a gel layer on the silica surface, silanol also goes through a condensation process. This could be attributed to open silica network which permits deep water penetration and result in degradation of the bioactive resin leading to calcium ion release [36, 37].

In harmony with this study results, Sajini et al. [2] compared remineralization and calcium ion release between ion-releasing composite, Giomer and GIC. Ion-releasing composite had the highest record, which support our study results. Also, the results are in accordance with Bhatia et al. [38] who revealed that ion-releasing composite releases significantly higher calcium ion than RMGI and attributed this to the differences in their composition, ion-releasing composite contains a flexible hydrophilic resin matrix that favors the release of ions. The results also in accordance with Kandil et al. [39] who reported that calcium ion release of ion-releasing composite was significantly higher than that of glass ionomer. Based on that EDX chemical analysis for calcium and phosphorus confirm the hydroxyapatite formation [22]. The outcome of this study revealed that all tested materials showed ability to resist demineralization. However, the three tested restorative materials showed significant differences in both calcium and phosphorus ion levels, and the rate of mineral release differed by time; accordingly, the null hypothesis was rejected.

The immediate scanning electron micrographs of Giomer showed large filler particles with different particle sizes [40], which is in agreement with the studies by Ruivo et al. [41] and Garoushi et al. [28] Therefore, the larger fillers of Giomer resulted in a rougher surface. Which may indicate good adaptation of Giomer / tooth interface with continuous interfaces between the restoration and tooth substrates. The good adaptation could be due to the use of a traditional two-step adhesive technique that involves removing the smear layer, unplugging the dentinal tubules, and demineralizing the dentin by acid etching, followed by the application of adhesive resin [19].

The micromorphology structure at the interface of Giomer show more dense particle and little voids than RMGI and ion-release composite, may be attributed to the high content of filler particles 83.3 wt% [30]. A relative degree of polymer matrix swelling can be observed in delayed Giomer scanning electron micrographs, according to Cimpean et al. [42] This may be attributed to the fact that Giomers with pre-reacted glassy surface polyacids regions have the ability to generate osmotic pressure, which may enhance water adsorption.

The Micromorphology structure at the interface of RMGI also showed intimate adaptation to the underlying tooth structure, which was in agreement with Tanumiharja et al. who reported that RMGI gave good adaptation to the underlying tooth structure and attributed that to conditioning with dentin conditioner [43]. Also, in accordance with Abdallah A et al. [19] who revealed good adaptation of RMGI and attributed this to RMGI containing resin, an unique bonding mechanism is expected. According to Lin et al. [44] mechanical interlocking may increase the RMGI stability by allowing resin monomer to penetrate the dentinal tubules.

Ion-releasing composite SEM revealed a crystal-like structure. Which was in agreement with Fahmy et al. [22] This morphology is frequently suggestive of the production of hydroxyapatite layers. Hence, the findings of previously mentioned studies support the outcome of current study and confirm the bioactivity [45]. According to the manufacturer’s recommendations, the use of a bonding agent was required [22]. Therefore; the current investigation used ion-releasing composite with pretreatment by bonding agent. The outcomes of EDX elemental analysis showed that there were significant differences in phosphorus and calcium level among the three different restorative systems at different times immediate and delayed, consequently, the null hypothesis was rejected.

All tested materials/ tooth interfaces revealed the development of a hybrid-like layer, and demonstrated interdiffusion zone or ion exchange layers which were more apparent after time of storage. Also, the interfaces showed unnoticeable changes after aging in delayed micrographs, which is a sign of the resistance to demineralization by crystallization and ion deposition (Figs. 2B and 3B, and 4B) [34].

One of the limitations of current study is that outcome cannot be generalized as other variables were not assessed; such as pH changes in the oral environment and stress initiated at cervical areas, may significantly affect ion release ability and bioactivity of restorative material. In addition, study of the effects of long-term aging is required to assess the long-term durability of restorations.

## Conclusion

Within the limitations of the current in-vitro study revealed that the ion-releasing capability of three tested restorative systems seems to be comparable. The rate of minerals release and diffusion are affected by time and composition. Ion-releasing composite seems to exhibit the highest remineralizations capability at tooth/ restoration interface.

## Electronic supplementary material

Below is the link to the electronic supplementary material.

Supplementary Material 1

Supplementary Material 2

## Data Availability

The datasets used and/or analyzed during the current study are available from the corresponding author on reasonable request.

## References

[1] Raghip AG, Comisi JC, Hamama HH, Mahmoud SH. In vitro elemental and micromorphological analysis of the resin-dentin interface of bioactive and bulk-fill composites. Am J Dent. 2023;36(1):3–7. 36917708

[2] Sajini SI, Alshawi BA, Alharbi LM. Assessment of remineralisation potentials of bioactive dental composite using an in-vitro demineralised dentine model. J Taibah Univ Med Sci. 2022;17(4):640–7. 35983447 10.1016/j.jtumed.2021.12.004 PMC9356364

[3] Kasraei S, Haghi S, Valizadeh S, Panahandeh N, Nejadkarimi S. Phosphate ion release and alkalizing potential of three Bioactive Dental materials in comparison with Composite Resin. Int J Dent. 2021;2021:5572569. 34040643 10.1155/2021/5572569 PMC8121605

[4] Imazato S, Kohno T, Tsuboi R, Thongthai P, Xu HH, Kitagawa H. Cutting-edge filler technologies to release bio-active components for restorative and preventive dentistry. Dent Mater J. 2020;39(1):69–79. 31932551 10.4012/dmj.2019-350

[5] Bhadra D, Shah NC, Rao AS, Dedania MS, Bajpai N. A 1-year comparative evaluation of clinical performance of nanohybrid composite with activa bioactive composite in Class II carious lesion: a randomized control study. J Conserv Dent. 2019;22(1):92–6. 30820090 10.4103/JCD.JCD_511_18 PMC6385569

[6] Francois P, Fouquet V, Attal JP, Dursun E. Commercially available fluoride-releasing restorative materials: a review and a proposal for classification. Mater (Basel) 2020, 13(10). 10.3390/ma13102313 PMC7287768 32443424

[7] Okuyama K, Murata Y, Pereira PN, Miguez PA, Komatsu H, Sano H. Fluoride release and uptake by various dental materials after fluoride application. Am J Dent. 2006;19(2):123–7. 16764137

[8] Naoum S, Ellakwa A, Martin F, Swain M. Fluoride release, recharge and mechanical property stability of various fluoride-containing resin composites. Oper Dent. 2011;36(4):422–32. 21819201 10.2341/10-414-L

[9] Itota T, Carrick TE, Yoshiyama M, McCabe JF. Fluoride release and recharge in giomer, compomer and resin composite. Dent Mater. 2004;20(9):789–95. 15451233 10.1016/j.dental.2003.11.009

[10] Omidi BR, Naeini FF, Dehghan H, Tamiz P, Savadroodbari MM, Jabbarian R. Microleakage of an enhanced Resin-Modified Glass Ionomer Restorative Material in primary molars. J Dent (Tehran). 2018;15(4):205–13. 30405729 PMC6218465

[11] Kaushik M, Yadav M. Marginal Microleakage properties of Activa Bioactive Restorative and Nanohybrid Composite Resin using two different adhesives in non Carious Cervical lesions - an in Vitro Study. J West Afr Coll Surg. 2017;7(2):1–14. 29951462 PMC6016748

[12] Kim T, Patel K, Comisi JC. Effect of SDF and SDF/KI treatment on microtensile bond strength of bioactive materials. Am J Biomed Sci Res. 2019;6(4):294–8.

[13] Sacher E, França R. Surface analysis techniques for dental materials. Dental Biomaterials. edn.: World Scientific; 2019. pp. 1–31.

[14] Scimeca M, Bischetti S, Lamsira HK, Bonfiglio R, Bonanno E. Energy Dispersive X-ray (EDX) microanalysis: a powerful tool in biomedical research and diagnosis. Eur J Histochem. 2018;62(1):2841. 29569878 10.4081/ejh.2018.2841 PMC5907194

[15] Breschi L, Mazzoni A, Ruggeri A, Cadenaro M, Di Lenarda R, De Stefano Dorigo E. Dental adhesion review: aging and stability of the bonded interface. Dent Mater. 2008;24(1):90–101. 17442386 10.1016/j.dental.2007.02.009

[16] Hamama HHH. Characterization of Bioactive Restoration/Dentine Interface. *Egyptian Dental Journal* 2019, 65(Issue 3 - July (Fixed Prosthodontics, Dental Materials, Conservative Dentistry & Endodontics)):2731–2738.

[17] Ebaya MM, Ali AI, Mahmoud SH. Evaluation of marginal adaptation and microleakage of three Glass Ionomer-based class V restorations: in Vitro Study. Eur J Dent. 2019;13(4):599–606. 31891976 10.1055/s-0039-3401435 PMC6938416

[18] De Caluwe T, Vercruysse CW, Ladik I, Convents R, Declercq H, Martens LC, Verbeeck RM. Addition of bioactive glass to glass ionomer cements: Effect on the physico-chemical properties and biocompatibility. Dent Mater. 2017;33(4):e186–203. 28196604 10.1016/j.dental.2017.01.007

[19] Abdallah A. Elemental and Micromorphological Analysis of New Alkasite Based Restorative Material/Tooth Interface. Egypt Dent J. 2022;68(1):1065–72.

[20] Hamama HHH. Effect of Dentine Surface Treatment on Bonding of Bioactive RMGI-based Restorative Material to Dentine. Egypt Dent J. 2019;65:2999–3006. Issue 3 - July (Fixed Prosthodontics, Dental Materials, Conservative Dentistry & Endodontics)).

[21] Boehm FC, Tanaka CJ. Restorative materials for restorations of non-carious cervical lesions: an overview. J Res Dentistry 2021, 9(4).

[22] Mohamed Fahmy M, Mosaa T, Abdelarouf R. Evaluation of Ion Release, Apatite formation and tooth-restoration interface of Bioactive Resin Composite Versus Conventional Resin Composite; an in Vitro Study. Egypt Dent J. 2021;67(2):1463–73.

[23] Reynolds EC. Calcium phosphate-based remineralization systems: scientific evidence? Aust Dent J. 2008;53(3):268–73. 18782374 10.1111/j.1834-7819.2008.00061.x

[24] Field J, Waterhouse P, German M. Quantifying and qualifying surface changes on dental hard tissues in vitro. J Dent. 2010;38(3):182–90. 20079800 10.1016/j.jdent.2010.01.002

[25] Thakur AK, Srivastava N, Chakrabarty T, Rebary B, Patidar R, Sanghavi RJ, Shahi VK, Ghosh PK. An improved protocol for electrodialytic desalination yielding mineral-balanced potable water. Desalination. 2014;335(1):96–101.

[26] Mazzaoui SA, Burrow MF, Tyas MJ. Fluoride release from glass ionomer cements and resin composites coated with a dentin adhesive. Dent Mater. 2000;16(3):166–71. 10762676 10.1016/s0109-5641(00)00003-8

[27] Kopperud SE, Tveit AB, Gaarden T, Sandvik L, Espelid I. Longevity of posterior dental restorations and reasons for failure. Eur J Oral Sci. 2012;120(6):539–48. 23167471 10.1111/eos.12004

[28] Garoushi S, Vallittu PK, Lassila L. Characterization of fluoride releasing restorative dental materials. Dent Mater J. 2018;37(2):293–300. 29279547 10.4012/dmj.2017-161

[29] Marovic D, Par M, Posavec K, Maric I, Stajdohar D, Muradbegovic A, Taubock TT, Attin T, Tarle Z. Long-Term Assessment of Contemporary Ion-releasing restorative Dental materials. Mater (Basel) 2022, 15(12). 10.3390/ma15124042 PMC9227571 35744101

[30] Kelic M, Kilic D, Kelic K, Sutej I, Par M, Peros K, Tarle Z. The Fluoride Ion Release from Ion-releasing Dental materials after Surface Loading by Topical Treatment with Sodium Fluoride Gel. J Funct Biomater 2023, 14(2). 10.3390/jfb14020102 PMC9958732 36826901

[31] Gonulol N, Ozer S, Sen Tunc E. Water Sorption, solubility, and Color Stability of Giomer Restoratives. J Esthet Restor Dent. 2015;27(5):300–6. 25145876 10.1111/jerd.12119

[32] Kelic K, Par M, Peros K, Sutej I, Tarle Z. Fluoride-releasing restorative materials: the Effect of a resinous coat on Ion Release. Acta Stomatol Croat. 2020;54(4):371–81. 33642601 10.15644/asc54/4/4 PMC7871432

[33] Alinda SD, Margono A, Putranto AW, Maharti ID, Amalina R, Rahmi SF. The comparison of Biofilm formation, mechanical and Chemical properties between Glass Ionomer Cement and Giomer. Open Dentistry J 2021, 15(1).

[34] Sobh EG, Hamama HH, Palamara J, Mahmoud SH, Burrow MF. Effect of CPP-ACP modified-GIC on prevention of demineralization in comparison to other fluoride-containing restorative materials. Aust Dent J. 2022;67(3):220–9. 35174511 10.1111/adj.12904

[35] Alebady MH, Hamama HH, Mahmoud SH. Effect of various surface coating methods on surface roughness, micromorphological analysis and fluoride release from contemporary glass ionomer restorations. BMC Oral Health. 2024;24(1):504. 38685036 10.1186/s12903-024-04234-5 PMC11057179

[36] Puspitasari D, Tajjalia N, Wibowo D, Wardhana AS. The Effect of Lactic Acid and Artificial Saliva Solution Immersion to the release of Calcium ions on Bioactive Resin. Dentino: Jurnal Kedokteran Gigi. 2021;6(2):190–4.

[37] Ruengrungsom C, Burrow MF, Parashos P, Palamara JEA. Evaluation of F, ca, and P release and microhardness of eleven ion-leaching restorative materials and the recharge efficacy using a new Ca/P containing fluoride varnish. J Dent. 2020;102:103474. 32941973 10.1016/j.jdent.2020.103474

[38] Bhatia K, Nayak R, Ginjupalli K. Comparative evaluation of a bioactive restorative material with resin modified glass ionomer for calcium-ion release and shear bond strength to dentin of primary teeth-an in vitro study. J Clin Pediatr Dent. 2022;46(6):25–32. 36624901 10.22514/jocpd.2022.022

[39] Kandil M, Sherief D. Marginal adaptation, compressive strength, water sorption, solubility and ion release of a claimed bioactive restorative material. Egypt Dent J. 2021;67:1–January. (Fixed Prosthodontics, Removable Prosthodontics and Dental Materials)).

[40] Colceriu Burtea L, Prejmerean C, Prodan D, Baldea I, Vlassa M, Filip M, Moldovan M, Moldovan ML, Antoniac A, Prejmerean V et al. New Pre-reacted Glass Containing Dental Composites (giomers) with Improved Fluoride Release and Biocompatibility. *Materials (Basel)* 2019, 12(23). 10.3390/ma12234021 PMC6926637 31816959

[41] Ruivo MA, Pacheco RR, Sebold M, Giannini M. Surface roughness and filler particles characterization of resin-based composites. Microsc Res Tech. 2019;82(10):1756–67. 31313442 10.1002/jemt.23342

[42] CIMPEAN S-I, AMBROSIE I, MOLDOVAN M, DELEAN A, PRODAN D, PREJMEREAN C, MOLDOVAN M, TOMOAIA-COTISEL M, COLCERIU-BURTEA L. TESTING OF NEW EXPERIMENTAL GIOMERS: WATER SORPTION, CONVERSION DEGREE, RADIOPACITY, MICROSTRUCTURE AND BIOLOGICAL BEHAVIOR. Stud Univ Babes-Bolyai Chem 2022, 67(1).

[43] Tanumiharja M, Burrow MF, Cimmino A, Tyas MJ. The evaluation of four conditioners for glass ionomer cements using field-emission scanning electron microscopy. J Dent. 2001;29(2):131–8. 11239588 10.1016/s0300-5712(00)00056-7

[44] Lin A, McIntyre NS, Davidson RD. Studies on the adhesion of glass-ionomer cements to dentin. J Dent Res. 1992;71(11):1836–41. 1401448 10.1177/00220345920710111401

[45] Ciobanu G, Carja G, Ciobanu O, Sandu I, Sandu A. SEM and EDX studies of bioactive hydroxyapatite coatings on titanium implants. Micron. 2009;40(1):143–6. 18242095 10.1016/j.micron.2007.11.011

