# Supplementary material for: Elemental analysis and micromorphological patterns of tooth/restoration interface of three ion-releasing class V restorations
Source: BMC Oral Health. 2024 Oct 15;24:1221. doi: 10.1186/s12903-024-04944-w (PMC11481381; doi:10.1186/s12903-024-04944-w)
Supplement: Supplementary file 1 — Supplementary Material 1 [file 12903_2024_4944_MOESM1_ESM.docx]

| Table (2) Two way ANOVA test for predicting P level | | | | | |
| --- | --- | --- | --- | --- | --- |
| Source | Type III Sum of Squares | df | Mean Square | F | p value |
| Corrected Model | 397.613^a^ | 5 | 79.523 | 304554.728 | .001* |
| Intercept | 1845.281 | 1 | 1845.281 | 7067034.574 | .001* |
| GP | 10.923 | 2 | 5.462 | 20916.447 | .001* |
| Time | .033 | 1 | .033 | 126.149 | .001* |
| GP * Time | 386.657 | 2 | 193.329 | 740407.298 | .001* |
| Error | .003 | 12 | .000 |  |  |
| Total | 2242.897 | 18 |  |  |  |
| Corrected Total | 397.616 | 17 |  |  |  |
| a. R Squared = 1.000 (Adjusted R Squared = 1.000) | | | | | |

Two Way ANOVA test was used to assess effect of changing in study group and time of assessment and the combination between 2 factors on P level . A statistically significant effect is detected for change in time and group and the combined effect of both factors ; 100 % of change in P level can be predicted by the combined effect of previous factors (R^2^ =1.0).

| **table (3) Post Hoc Tukey test for studying effect of subgroup comparison (paired comparison))** | | | | | | |
| --- | --- | --- | --- | --- | --- | --- |
| P  Tukey HSD |  |  |  |  |  |  |
| (I) GP | (J) GP | Mean Difference (I-J) | Std. Error | p value | 95% Confidence Interval | |
|  |  |  |  |  | Lower Bound | Upper Bound |
| Ion-releasing composite | Giomer | 1.8883^*^ | .00933 | .001* | 1.8634 | 1.9132 |
|  | RMGI | 1.1817^*^ | .00933 | .001* | 1.1568 | 1.2066 |
| RMGI | Ion-releasing composite | -1.8883^*^ | .00933 | .001* | -1.9132 | -1.8634 |
|  | Giomer | -.7067^*^ | .00933 | .001* | -.7316 | -.6818 |
| Giomer | Ion-releasing composite | -1.1817^*^ | .00933 | .001* | -1.2066 | -1.1568 |
|  | RMGI | .7067^*^ | .00933 | .001* | .6818 | .7316 |

Table (3) :Post Hoc Tukey test was used to assess pairwise for Phosphorus comparison between subgroups and demonstrates that between Ion-releasing composite & Giomer (p=0.001) , between Ion-releasing composite & RMGI (p=0.001) and between RMGI & Giomer (p=0.001)

| Table (4) Two way ANOVA test for predicting ca level | | | | | |
| --- | --- | --- | --- | --- | --- |
| Source | Type III Sum of Squares | df | Mean Square | F | p value |
| Corrected Model | 215.011^a^ | 5 | 43.002 | 157967.629 | .001* |
| Intercept | 11121.867 | 1 | 11121.867 | 4.086E7 | .001* |
| GP | 144.074 | 2 | 72.037 | 264626.347 | .001* |
| Time | 8.419 | 1 | 8.419 | 30925.735 | .001* |
| GP * Time | 62.518 | 2 | 31.259 | 114829.857 | .001* |
| Error | .003 | 12 | .000 |  |  |
| Total | 11336.882 | 18 |  |  |  |
| Corrected Total | 215.015 | 17 |  |  |  |
| a. R Squared = 1.000 (Adjusted R Squared = 1.000) | | | |  |  |

Two Way ANOVA test was used to assess effect of changing in study group and time of assessment and the combination between 2 factors on Ca level . A statistically significant effect is detected for change in time and group and the combined effect of both factors ; 100 % of change in Ca level can be predicted by the combined effect of previous factors (R^2^ =1.0).

| **Table (4) Post Hoc Tukey test for studying effect of subgroup comparison (paired comparison))** | | | | | | |
| --- | --- | --- | --- | --- | --- | --- |
| (I) GP | (J) GP | Mean Difference (I-J) | Std. Error | p value | 95% Confidence Interval | |
|  |  |  |  |  | Lower Bound | Upper Bound |
| Ion-releasing composite | Giomer | -6.3917^*^ | .00953 | .001* | -6.4171 | -6.3663 |
|  | RMGI | -5.5150^*^ | .00953 | .001* | -5.5404 | -5.4896 |
| RMGI | Ion-releasing composite | 6.3917^*^ | .00953 | .001* | 6.3663 | 6.4171 |
|  | Giomer | .8767^*^ | .00953 | .001* | .8513 | .9021 |
| Giomer | Ion-releasing composite | 5.5150^*^ | .00953 | .001* | 5.4896 | 5.5404 |
|  | RMGI | -.8767^*^ | .00953 | .001* | -.9021 | -.8513 |

Table (3) : Post Hoc Tukey test was used to assess pairwise for calcium comparison between subgroups and demonstrates that between Ion-releasing composite & Giomer (p=0.001) , between Ion-releasing composite & RMGI (p=0.001) and between RMGI & Giomer (p=0.001)
