# Supplementary material for: Elemental analysis and micromorphological patterns of tooth/restoration interface of three ion-releasing class V restorations
Source: BMC Oral Health. 2024 Oct 15;24:1221. doi: 10.1186/s12903-024-04944-w (PMC11481381; doi:10.1186/s12903-024-04944-w)
Supplement: Supplementary file 2 — Supplementary Material 2 [file 12903_2024_4944_MOESM2_ESM.docx]

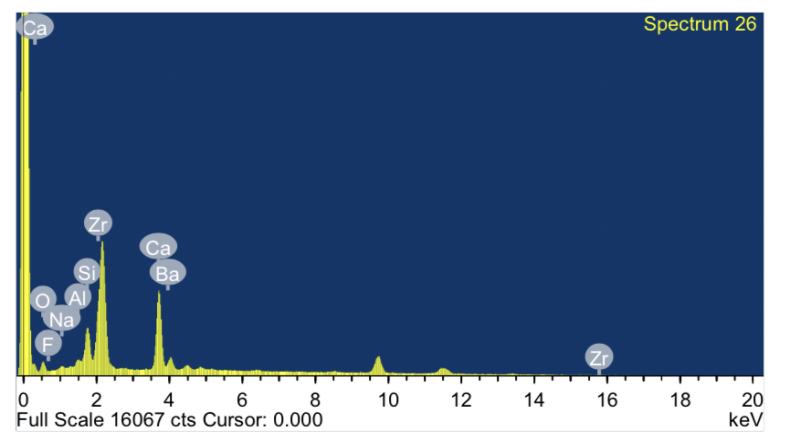

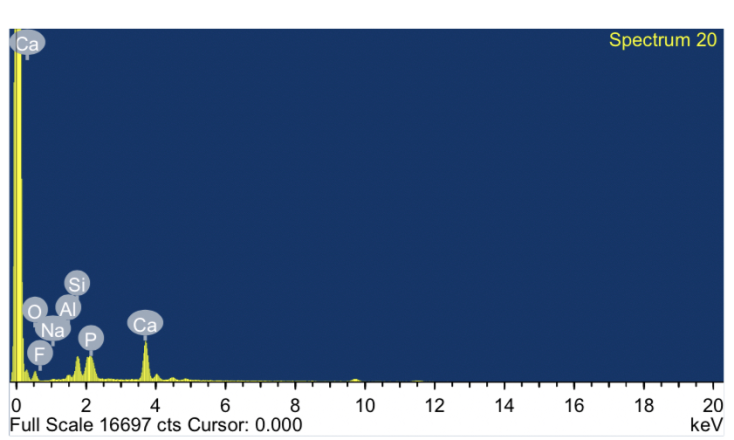

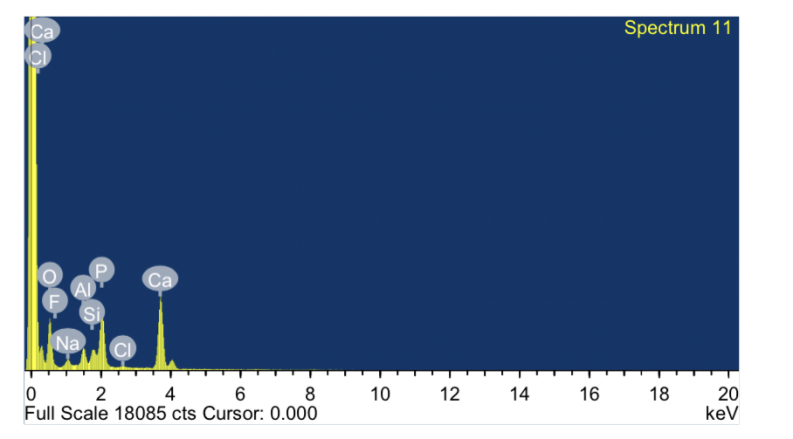

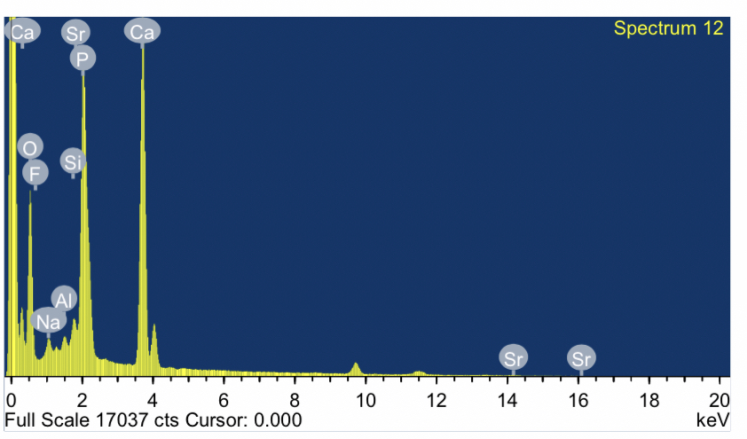

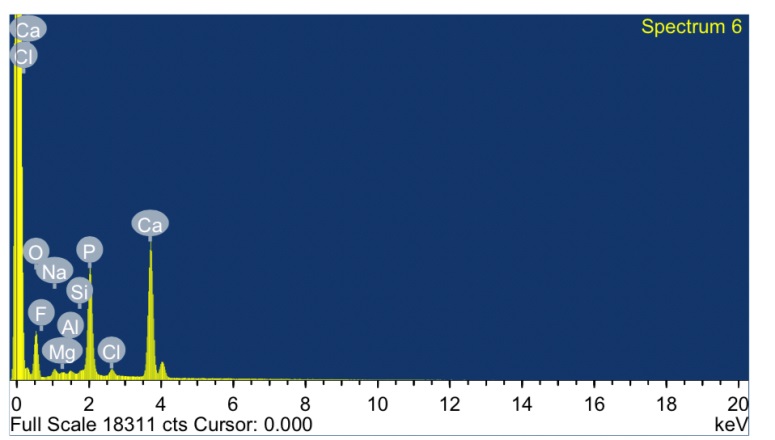

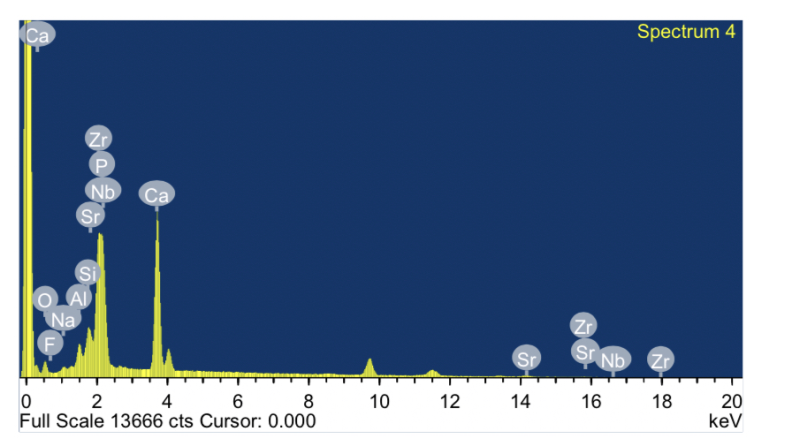
**Figure2.** EDX elemental analysis at tooth/restoration interface (A) Giomer immediate analysis, (B) Giomer delayed analysis, (c) ion-releasing composite immediate analysis, (D) ion-releasing composite delayed analysis, (E) RMGI immediate analysis, (F) RMGI delayed analysis.

**C**

**F**

**E**

**D**

**B**

**A**


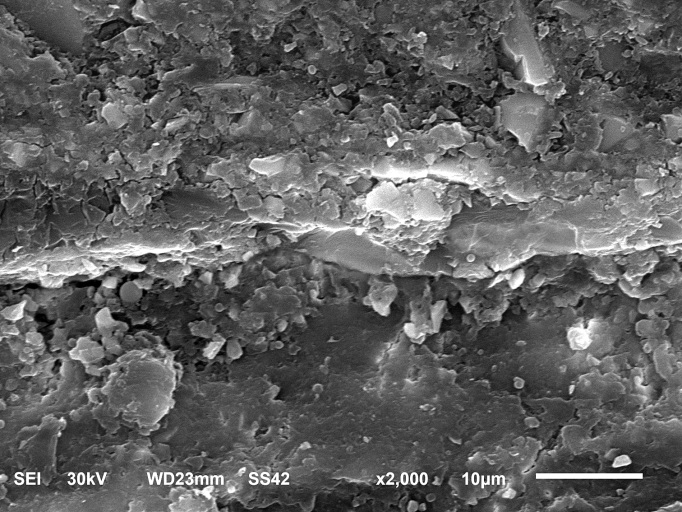

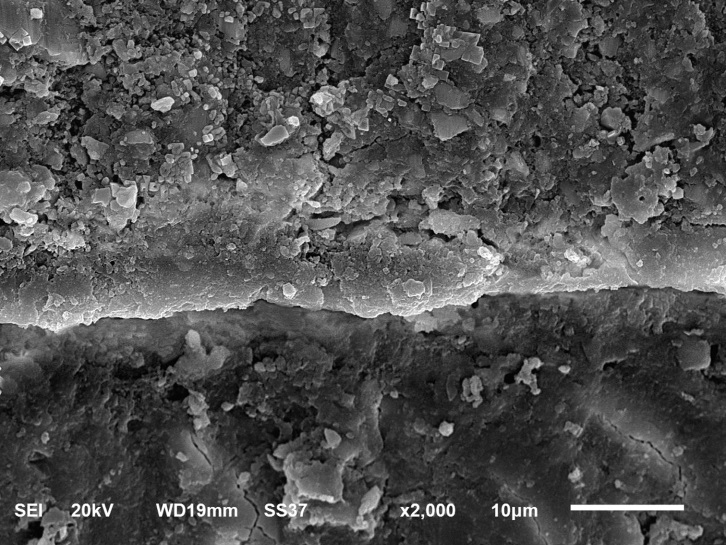


B

A

**Giomer**

**Giomer**

**Tooth**

**Tooth**

**Figure3.** SEM micrography showing micromorphology of tooth/ giomer interface: (A) immediate scanning, (B) delayed scanning


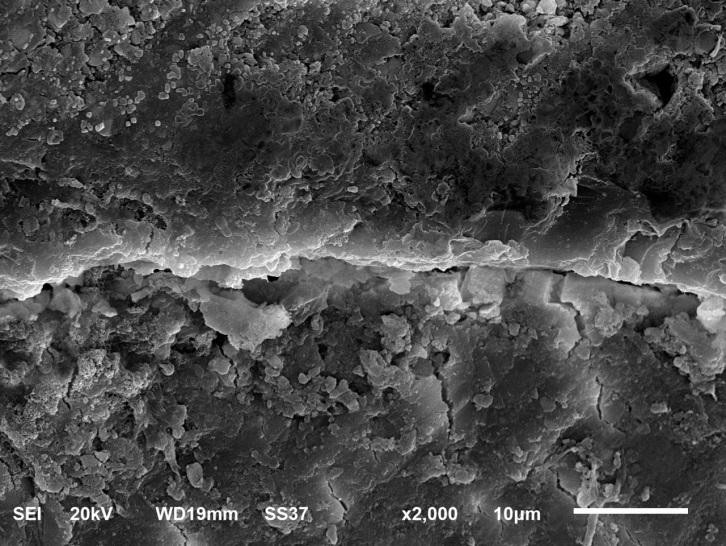

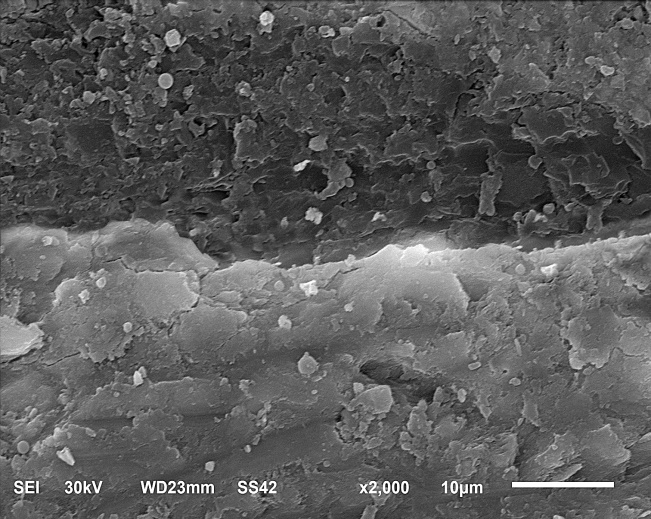


A

B

**Ion-releasing composite**

**Ion-releasing composite**

**Tooth**

**Tooth**

**Figure4.** SEM micrography showing micromorphology of tooth/ ion-releasing composite interface: (A) immediate scanning, (B) delayed scanning (the arrows pointing to crystal-like structure)


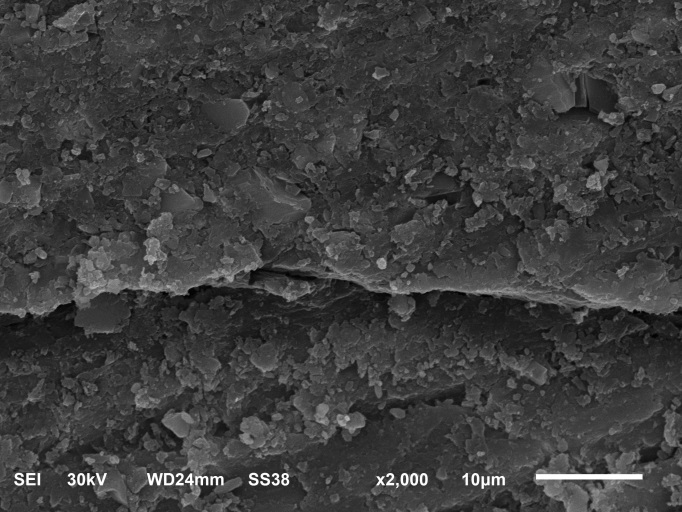

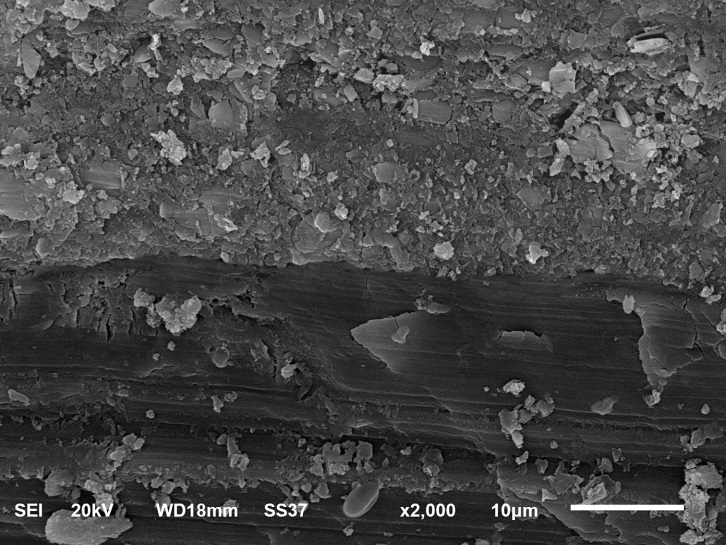


**RMGI**

B

A

**RMGI**

**Tooth**

**Tooth**

**Figure5.** SEM micrography showing micromorphology of tooth/ RMGI interface: (A) immediate scanning, (B) delayed scanning.
